# Comparison of early season crop types for wheat production and nitrogen use efficiency in the Jianghan Plain in China

**DOI:** 10.7717/peerj.11189

**Published:** 2021-04-09

**Authors:** Rui Yang, Ke Liu, Shiying Geng, Chengxiang Zhang, Lijun Yin, Xiaoyan Wang

**Affiliations:** 1 College of Agriculture, Yangtze University, Jingzhou, China; 2 College of Agronomy and Biotechnology, China Agricultural University, Beijing, China; 3 Engineering Research Center of Ecology and Agricultural Use of Wetland, Yangtze University, Jingzhou, China

**Keywords:** Crop rotation, Rice wheat cropping system, Grain yield, Nitrogen use efficiency

## Abstract

The rice-wheat (RW) cropping system is one of the most prevalent double-cropping systems used to farm the Jianghan Plain in China. However, it can lead to low wheat yields and reduced nitrogen use efficiency compared with dryland wheat (DW). We evaluated wheat yield and nitrogen use efficiency for two rotations (summer rice-winter wheat and summer soybean-winter wheat) from 2017 to 2019 and applied the results to improve nitrogen management for planting wheat after rice in the Jianghan Plain. Field experiments were conducted over two years with two nitrogen treatments: traditional nitrogen management (M1: 90 kg N ha^−1^ was applied at sowing and jointing, respectively ) and optimized nitrogen management (M2: 60 kg N ha^−1^ was applied at sowing, wintering and jointing, respectively). The highest total wheat production was achieved under M2 for both cropping systems and the two-year average yield was 6,128 kg ha^−1^ in DW and 6,166 kg ha^−1^ in RW. The spike number in DW was 15% higher than RW in M1 and 13% higher in M2, but the kernel per spike and 1,000-grain weight was lower than RW. The nitrogen accumulation of DW was 24% higher than RW in M1 and 33% in M2. Compared with RW, DW had higher NO_3_^−^ content in the soil surface layer (0–20 cm) and a higher root length density (RLD) in the deeper layer (40–60 cm), which may account for the higher N uptake in DW. Our results show that the grain yield of RW was comparable to that of DW by optimum nitrogen management. The rice-wheat cropping system combined with optimum nitrogen management may be of economic and agronomic benefit to the wheatbelt in the Jianghan Plain in China.

## Introduction

Winter wheat accounts for approximately 95 percent of China’s total wheat output. More than 75 percent of the wheat area is planted using a multi-cropping system in which two or more crops can be planted and harvested each year in sequential cropping or relay cropping. The middle and lower Yangtze River Basin is a highly productive agricultural region and the rice-wheat rotation system is used predominantly (Liu et al., 2016). Three provinces (Hubei, Anhui and Jiangsu) account for 58% of the rice–wheat rotation area in China (Han et al., 2020). However, winter wheat exhibits low productivity due to sub-optimal varieties, and nitrogen (N) and waterlogging stress (Ding et al., 2020). Farmers have resorted to using basal loads of higher N fertilizers to maintain high yields (Cui, Chen & Zhang, 2010; Tian et al., 2020). The current fertilizer use has already reached a very high level, and further increased N is not likely to increase yields. Therefore, it is very important to develop alternative nutrient management practices for the rice-wheat cropping system.

The average yields in dryland wheat cropping zones are generally higher than rice-wheat cropping in the Jianghan Plain, where wheat production is dependent up on precipitation (Che et al., 2016). The yield gap may be narrowed by optimizing nitrogen and adopting of advanced crop varieties. For example, the yield of winter wheat in the rice-wheat cropping system was comparable to the maize-wheat cropping system when N was 210 kg ha^−1^(Shi et al., 2012; Hartmann et al., 2015). The majority of studies were conducted in different years or regions (e.g., the first year was dedicated to the dryland wheat experiment and the second year to the rice-wheat experiment). Yield and N use efficiency are complex traits that are subject to strong interactions between the genotype, environment, and field management and it remains uncertain whether the recommendations for N management would be useful under different environments. The extent to which wheat yield is influenced by the timing and application of N, relative to crop phenology, is unknown under the rice-wheat rotation system in the Jianghan Plain in China.

Excessive N application may lead to low nitrogen use efficiency (NUE) despite of the finding that N fertilization may increase grain yield (Ju et al., 2007; Kaur, Kaur & Asthir, 2017). Ju et al. (2007) found that excessive rainfall in the rice-wheat cropping systems generally leads to a low NUE. The N content of grains in dryland wheat was 170–200 kg ha^−1^, which was higher than rice-wheat systems (120–140 ka ha^−1^) at the same yield level (Li et al., 2016; Zhang et al., 2017). The root is responsible for N uptake, and root morphology and distribution determine a plant’s ability to absorb N (Jiang et al., 2017). Tian et al. (2020) observed that the surface soil layer (0–20 cm) accounted for over three quarters the total root-weight density in the rice-wheat cropping system. However, dryland wheat in the North China Plain has a higher proportion of roots in the deep soil layer than those found in rice-wheat cropping (Liu et al., 2018). The uptake of N by wheat is also affected by the effective soil N content. Under the same fertilization conditions, the accumulation of nitrate nitrogen in dryland wheat is higher than that in the rice-wheat cropping system (Tian et al., 2020; Zheng et al., 2020). This may be due to the decreased accumulation of nitrate N with an increased soil water content. The middle and lower levels of the Yangtze River have abundant rainfall and shallow groundwater levels, leading to high rates of N loss as a result of leaching. However, the factors influencing the N uptake of wheat in the two systems ought to be verified in the same region and year.

We conducted a two-year field experiment with two rotation crops: summer soybean-winter wheat (DW) and summer rice-winter wheat (RW) under different N management strategies. We sought to compare the differences in the yield and yield component of DW and RW and to identify the reasons for the differences in nitrogen uptake between DW and RW.

## Material and Methods

### Experimental site

**Figure 1:**
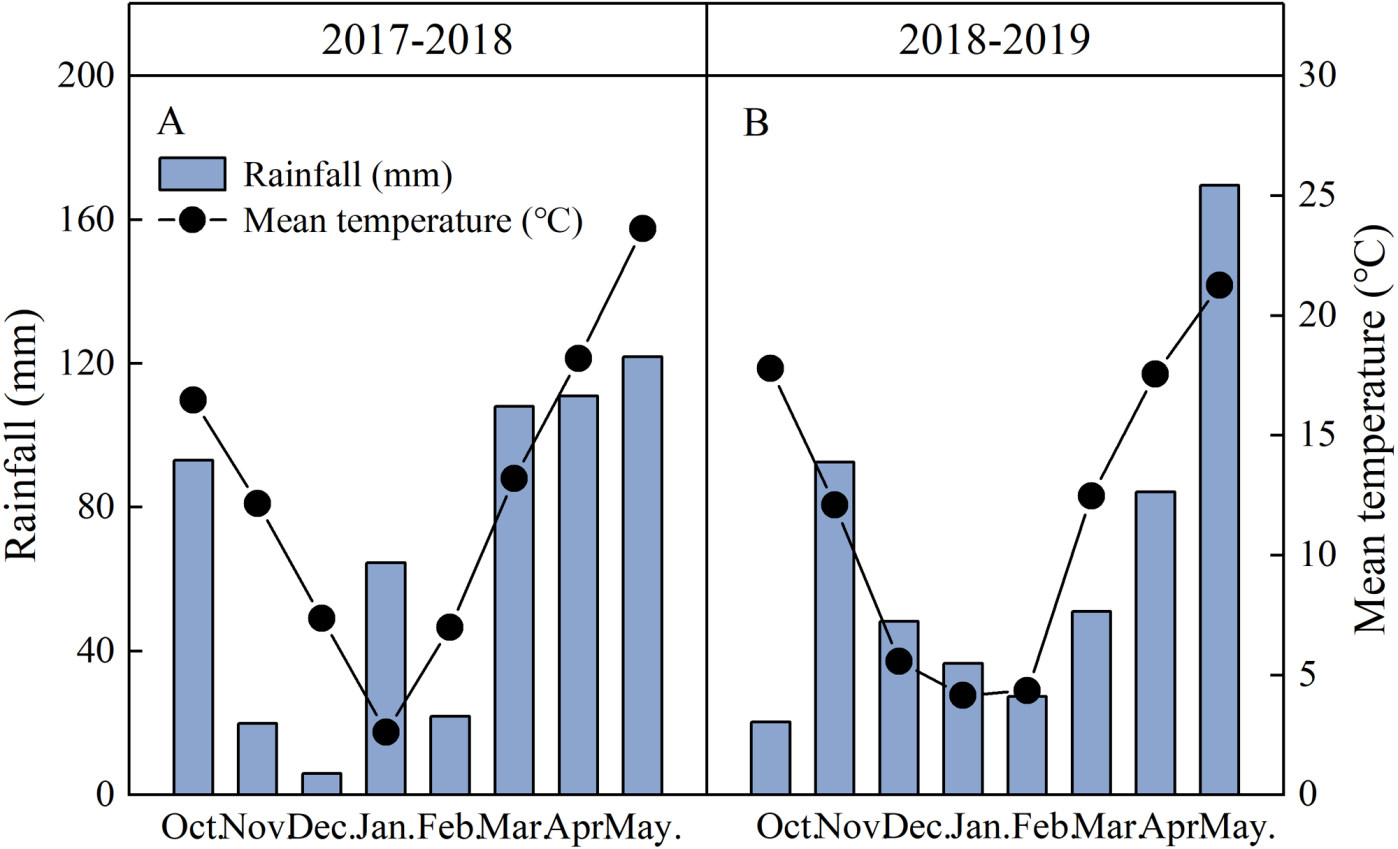
Monthly total rainfall and monthly mean temperatures recorded on-site in 2017–2019 wheat-growing seasons in Jingzhou, Hubei Province, China.

Field experiments were conducted at the experimental farm of Yangtze University (112°08′E, 30°36′N) in the Jianghan Plain, Hubei Province, China from 2017 to 2019. Our experiment was conducted with two crop rotations: summer soybean-winter wheat (DW) and summer rice-winter wheat (RW). The study region experiences abundant rainfall with total of 546 mm of rain during the wheat-growing season of 2017–2018, and mean air temperature of 12.6 °C. There were 530 mm of rain that fell during the wheat season in 2018–2019, and the mean air temperature was 11.9 °C (Fig. 1). Meteorological data were recorded using an automatic weather station located near the experimental site. Soil samples were air-dried at 20 cm before the experiment, and all parameters were measured according to conventional laboratory methods (Bao, 2000). The soil parameters of the two wheat cropping systems are shown in Table 1.

**Table 1:** The soil fertility status of the tested soil (0–20 cm).

cropping systems	pH	Organic matter (g kg^−1^)	Available nitrogen (mg kg^−1^)	Available phosphorous (mg kg^−1^)	Available potassium (mg kg^−1^)
DM	7.80	11.00	82.03	15.20	51.11
RW	7.79	12.37	51.22	12.07	52.74

### Experimental design

We cultivated ZhengMai 9023 (Pedigree: Xiaoyan6/Xinong65) for our study, which is a common high-yielding wheat cultivar in the Jianghan Plain. The experiment was designed as a randomized complete block and each plot measured 2 ×6 m, with three replications. Wheat seeds were sown by hand into the plots at a sowing rate of 125 kg ha^−1^. The nitrogen treatments are shown in Table 2. N fertilizer was applied in the form of urea (46% N) that was ploughed into the soil during sowing and was spread as top-dressing. Plots were supplied with P (105 kg P_2_O_5_ ha^−1^, calcium superphosphate) and K (105 kg K_2_O ha^−1^ potassium sulfate) fertilizer during the sowing period (10 November in 2017 and 1 November in 2018) in the two wheat-growing seasons. We did not need to irrigate during the two growing seasons due to the abundant rainfall. Herbicides, pesticides, and fungicides were sprayed according to standard growing practices to avoid yield loss.

**Table 2:** Nitrogen application time and amount used in different N treatments.

N treatment	Sowing (kg N ha^−1^)	Wintering (kg N ha^−1^)	Jointing (kg N ha^−1^)	Total N amount (kg N ha^−1^)
CK	0	0	0	0
M1	90	0	90	180
M2	60	60	60	180

### Sampling and measurements

#### Grain yield and yield components

We selected mature plants from a 2 m^2^ harvest area in the middle of each plot to determine the grain yield and yield components. The spike number was enumerated in each plot and recorded prior to harvest. All spikes were manually harvested, threshed, and weighed to calculate grain yield. One thousand random kernels from each harvested grain were weighed to calculate a 1,000-kernel weight. Grain moisture was measured using a grain analyzer (InfratecTM, Foss, Denmark). Grain yield and 1,000-kernel weight were adjusted to 13% moisture. The average number of kernels per spike was enumerated from 30 spikes.

### Nitrogen content

The shoot biomass was harvested from twenty mature plants in each plot and separated into stem and sheath, flag leaf, other leaves, hull and rachis and grain. Samples were oven dried at 65 °C for at least 48 hr until they were a constant weight. Nitrogen concentrations in plant samples were determined by the Kjeldahl method (Hibbard, 1910). The plant’s uptake of N was calculated by multiplying the concentration of N in plants by the dry biomass to give N accumulation.

### Root measurement

Root measurements were tested following the protocol described by Liu et al. (2018). Root measurements were made using the CI-600 root growth monitoring system (CID Bio-Science-CI-600, Camas, WA, USA) fitted with a scanner head for collecting images, a laptop computer, and 1 m standard clear soil tubes (50.8 mm internal diameter) with end caps. An auger of the same external diameter as the tube was used to facilitate close tube soil contact. The scanner was inserted into each tube a depth of 85 cm. Minirhizotron tubes were inserted into the soil of the central sowing line of each plot before sowing. The above-ground part of each tube was covered with thermal insulation foils to prevent light, condensation, and sun warming of the tube. Images were captured at three depths with the aid of an automatic indexing handle equivalent (given the angle of the tube at 45° off vertical) to 0–20, 20–40, and 40–60 cm. Each scan provided a nearly 360° image (21.59 × 19.56 cm) with a resolution of 200 dpi. Images were captured at maturity. Root length density (RLD) per sample and tube segments for each plot were calculated from these images using WinRhizotron^®^ software.

### Measurement of nitrate-nitrogen content of soil

We collected soil samples from the 0–200 cm soil layer by soil drilling and electric earth picking in each district from mature wheat samples collected in 2018. Samples were taken from 2 sampling points in each plot, and samples were taken from a soil layer every 20 cm. The samples at 2 sampling points were mixed evenly and stored in a −20 °C freezer for future use. The sample treatment and determination steps were as follows: after thawing, 10 g of a fresh soil sample were taken and put it into a 100 mL trigonometric bottle, 0.1 g of calcium sulfate and 50 ml of water were added and shaken for 30 min. The mixture was allowed to stand for 5 min and the supernatant was filtered for later use. A SMARTCHEN automatic intermittent chemical analyzer was used to measure the soil nitrate content, and the residual nitrogen accumulation of 0–200 cm soil was calculated according to the soil bulk density of each layer. Soil bulk density was determined by the cutting ring method. The water content of the soil was determined by drying the soil samples in an oven at 105 °C until a constant weight.

### Measurement of soil moisture

**Figure 2:**
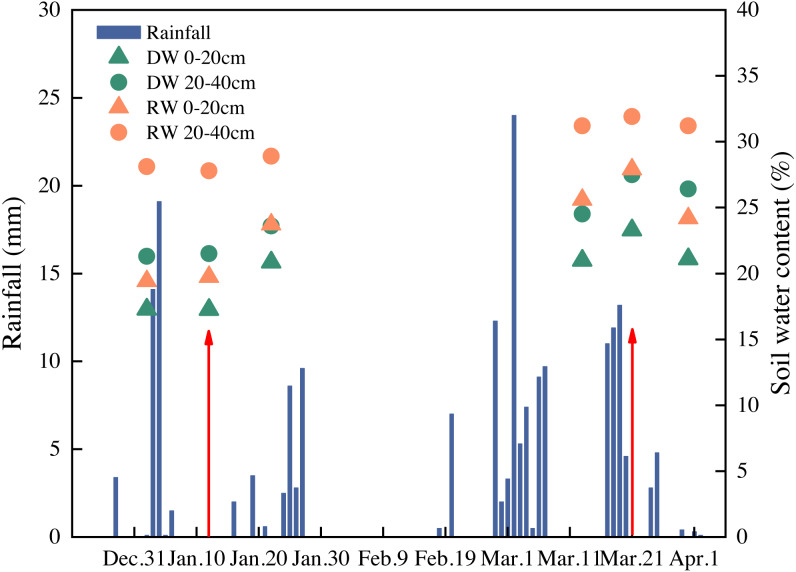
The soil moisture content and daily precipitation. Red arrows represent timing of nitrogen application (only M2 topdressed on 12 Jan; M1 and M2 topdressed on 21 Mar).

Five separate holes were drilled using a soil drill in each plot to obtain soil-moisture measurements at different soil depths. Samples were obtained on 2, 12, and 22 January and 13, 21, and 20 March, respectively. The soil water content was determined by drying the soil samples in an oven at 105 °C until at a constant weight. The soil moisture content at the 0–20 and 20–40 cm soil layers is shown in Fig. 2. Generally, the soil moisture in RD was higher than DW across different soil layers throughout the whole growth season.

### Statistical analysis

Data were analyzed using three-way (year, cropping system and nitrogen management) analysis of variance with SAS 9.2 (SAS Institute, Cary, NC). The means of treatment were compared using the least significant difference (LSD) at *P* < 0.05.

## Results

### Yield and yield components

The interactive effects of year (Y) × cropping system (C) × nitrogen management (M) were significant for grain yield. Both grain yield and kernel per spike were significantly affected by the interaction of C ×M and Y ×M. Y, C, and M had a significant effect on all yield components. There was minor yield gap between DW and RW under M2, and both reached the highest grain yield under M2 over two years. The average grain yield increased by 11% in M2 in DW and 15% in RW in both years compared with M1. The spike number in M1 was comparable with M2 for yield components but kernel per spike in M1 was significantly lower than M2 regardless of cropping system. The mean spike numbers in DW were 15% higher than RW in M1 and 13% in M2. The opposite trend was observed in kernel per spike. The kernel per spike in DW was 6% lower than RW in M1 and 4% in M2. There was no difference in the 1,000-kernel weight between M1 and M2 but the 1,000-kernel weight in DW was significantly lower than RW in 2018–2019.

### Nitrogen accumulation in aboveground organs in wheat maturation

**Figure 3:**
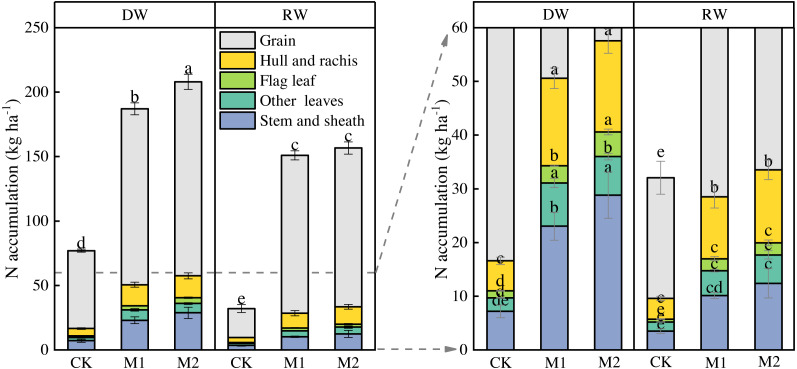
Effects of nitrogen management on nitrogen accumulation (kg ha^−1^) in organs of two wheat cropping systems at maturity.

Increasing the nitrogen supply significantly increased nitrogen accumulation in aboveground organs in both cropping system (Fig. 3). DW had the highest nitrogen accumulation in M2, but there was no significant difference in nitrogen accumulation between M1 and M2 in RW. The nitrogen accumulation in the aboveground organs of DW at maturity was significantly higher than that of RW, regardless of nitrogen treatments. The nitrogen accumulation of DW was 24% higher than RW in M1 and 33% in M2. Nitrogen accumulation of grain samples was highest in both DW and RW. The biggest difference was found in nitrogen accumulation in grain between DW and RW, followed by nitrogen accumulation in hull and rachis. Nitrogen accumulation of grain in DW was 11% higher than RW in M1, and 22% in M2.

### The root length density (RLD) distribution at 0–60 cm soil layer

**Figure 4:**
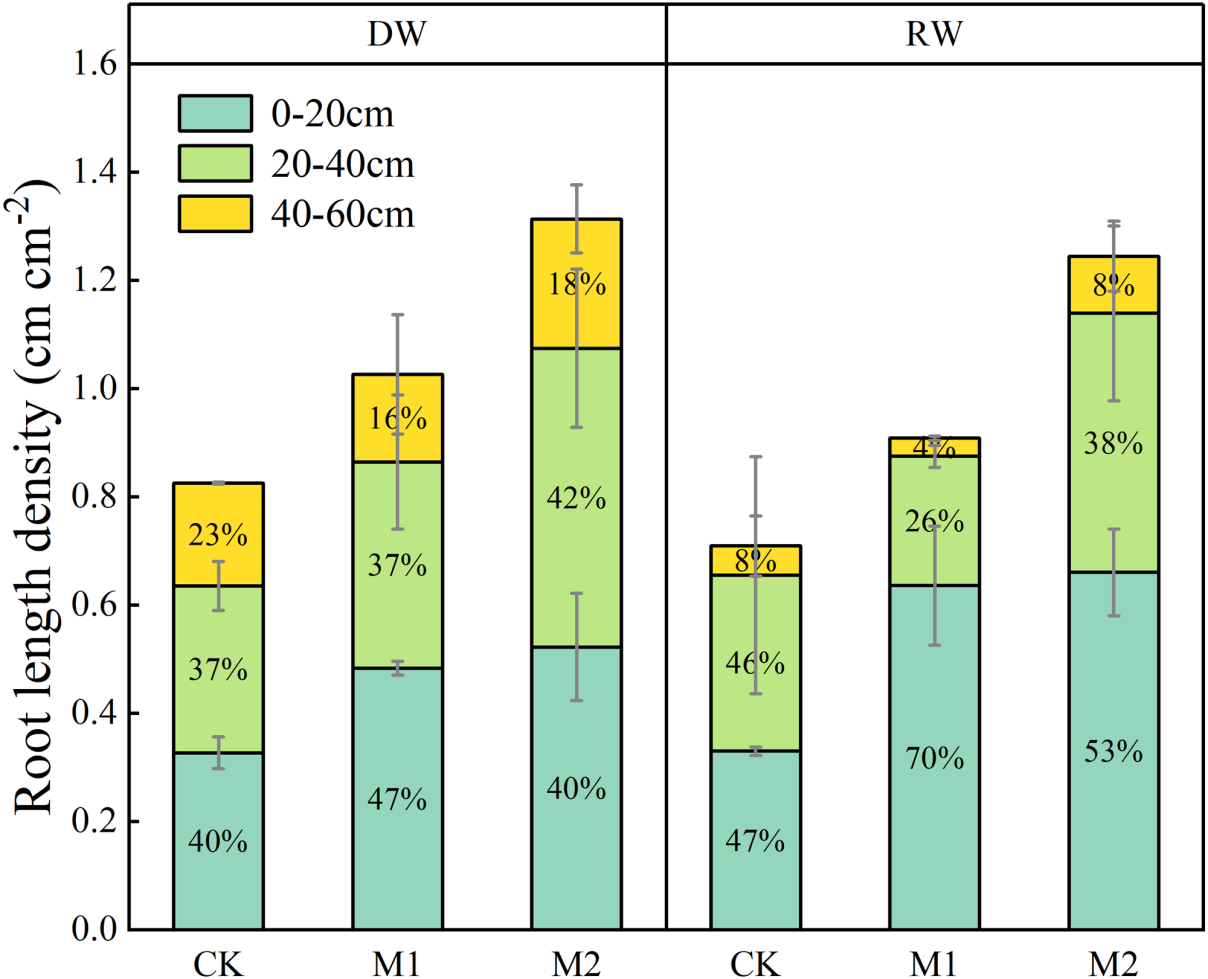
Effects of nitrogen management on root length density (RLD) distribution of two wheat cropping systems at maturity.

Increasing the nitrogen supply timing increased root length density (RLD) in both cropping systems (Fig. 4). The distribution in RLD at soil depths were different between RW and DW, especially in deeper soil. The surface soil layer (0–20 cm) accounted for over half the total RLD in both RW and DW but there were few roots (8%) in the deep soil profile in RW. However, DW had a relatively higher RLD (*ca.* 16–18%) in the deep soil profile, although its RLD in surface soil layer was lower than RW.

### Distribution and accumulation of nitrate nitrogen content in 0–200 cm soil

#### Distribution of soil nitrate nitrogen content

**Figure 5:**
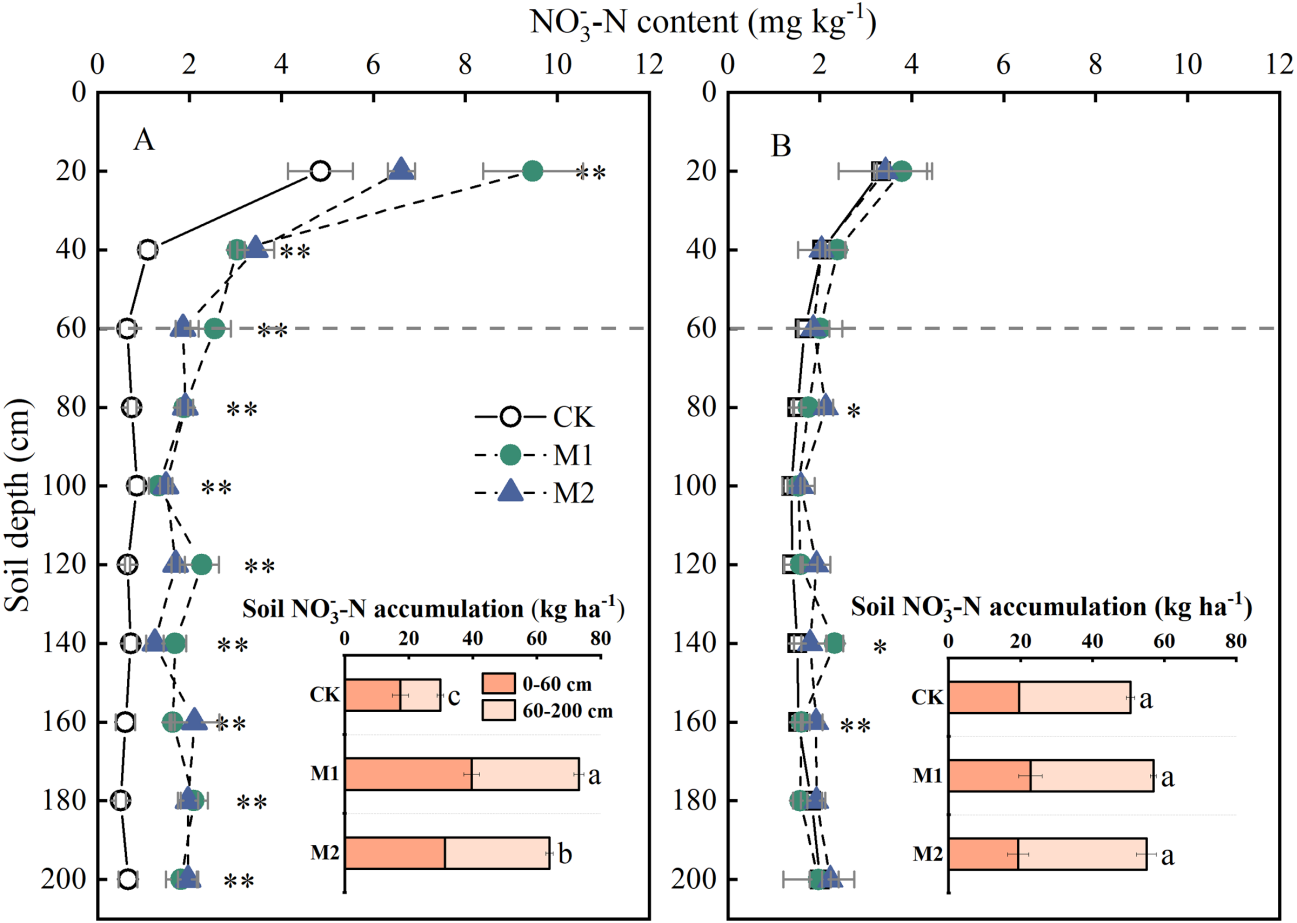
Effects of nitrogen management on NO${}_{3}^{-}$-N content (mg kg^−1^) and accumulation (kg ha^−1^) in 0–2 m soil of dryland wheat (A) and rice wheat (B) cropping systems at maturity.

We observed significant differences in the soil nitrate nitrogen content (0–60 cm) in DW but the differences were not significant in RW (Fig. 5). The two cropping systems showed that the 0–20 cm soil layer had the highest nitrate nitrogen content in M1, with a nitrate nitrogen content of 9.5 mg kg^−1^(DW) and 3.8 mg kg^−1^(RW), respectively. Nitrate nitrogen content in M1 was 43% higher than M2 but nitrate nitrogen content was similar under the two treatments in RW. Nitrate nitrogen content gradually decreased with the depth of the soil layer.

#### Soil nitrate accumulation

Increasing the nitrogen supply significantly increased the nitrate nitrogen accumulation of the soil (0–200 cm) in DW, but there was no significant difference in RW (Fig. 5). The nitrate nitrogen accumulation of the 0–60 cm soil layer in DW was 75% higher than RW in M1 and 63% in M2. An opposite trend was observed in the nitrate nitrogen accumulation of the 60–200 cm soil layer. The nitrate nitrogen accumulation of the 60–200 cm soil layer in DW was 2% lower than RW in M1 and 9% in M2.

## Discussion

The yield and N uptake of wheat in rice-wheat (RW) and soybean-wheat (DW) rotation cropping systems were compared and analyzed under different nitrogen management systems. Our results showed that RW combined with appropriate nitrogen management could achieve similar yields as DW (Table 3). We analyzed the variation rules of the root system, nitrate nitrogen, and nitrogen uptake under different nitrogen management systems as well as the main reasons for the differences in nitrogen uptake.

**Table 3:** Effects of nitrogen management on grain yield and yield composition of two wheat cropping systems.

Year (Y)	Cropping system (C)	Nitrogen management (M)	Grain yield	Spike number	Kernel Per spike	1,000-kernel weight
2017–2018	DW	CK	3,755 ± 610c	317 ± 12c	29.7 ± 1.7d	40.4 ± 0.2a
	DW	M1	5,787 ± 88b	477 ± 10a	35.0 ± 0.4c	39.1 ± 0.9a
	DW	M2	6,418 ± 114a	481 ± 15a	38.5 ± 0.8b	39.2 ± 0.5a
	RW	CK	1,254 ± 252d	293 ± 17d	18.9 ± 2.4e	39.6 ± 1a
	RW	M1	5,688 ± 127b	451 ± 6b	37.1 ± 0.4b	40.4 ± 0.5a
	RW	M2	6,559 ± 379a	459 ± 14b	39.4 ± 0.9a	40.7 ± 0.4a
2018–2019	DW	CK	1,945 ± 329c	274 ± 12c	22.0 ± 2.8d	40.7 ± 0.8c
	DW	M1	5,217 ± 110b	446 ± 30a	32.3 ± 0.7c	40.8 ± 0.5c
	DW	M2	5,838 ± 108a	456 ± 12a	35.1 ± 0.6ab	41.8 ± 0.6bc
	RW	CK	1,413 ± 342d	225 ± 6d	20.9 ± 1.4d	42.7 ± 1.2ab
	RW	M1	5,056 ± 288b	362 ± 22b	34.6 ± 1b	42.9 ± 1.3ab
	RW	M2	5,774 ± 72a	373 ± 23b	36.9 ± 0.6a	43.8 ± 1.4a
*ANOVA*						
Y(Year)			55.45^**^	132.36^**^	50.93^**^	65.81^**^
C (Cropping system)			32.26^**^	89.7^**^	4.15^*^	22.31^**^
M (Nitrogen management)			701.93^**^	498.42^**^	511.47^**^	1.76ns
Y ×C			9.05^**^	21.62^**^	20.36^**^	5.26^*^
Y ×M			0.48ns	0.11ns	0.06ns	1.37ns
C ×M			27.26^**^	1ns	42.31^**^	1.23ns
Y ×C ×M			13.84^**^	1.53ns	14.9^**^	1.52ns

**Notes.**

Values followed by different lowercase letters within a column are significantly different at *P* < 0.05.

NS not significant

* Significant at the 0.05 probability level.

** Significant at the 0.01 probability level.

### Effects of cropping systems on yield and nitrogen uptake

We observed similar yields of wheat in DW and RW, but the mean spike numbers in DW was 15% higher than RW in M1 and 13% in M2 (Table 3). This may be due the difference in soil textures between the soybean-wheat and rice-wheat cropping systems. The direct planting of wheat without tillage is encouraged in the rice-wheat cropping system. Soil compaction after mechanical rice harvesting increases its bulk density and reduces water infiltration and nutrient availability compared with the soil texture of soybean-wheat rotation soil. Compacted soil inhibits the emergence of shoots (Timsina & Connor, 2001). As a result, the population of RW under the same sowing density was lower than DW. However, a lower population may allow for wider plant spacing, which impacts the leaf area, light interception, and canopy apparent photosynthesis (Tao et al., 2018). This leads to the production of more photosynthetic products that may significantly increase the spike number and kernel number, compensating for detrimental effects of lower population on other yield components to some degree (Liu et al., 2020). Ensuring early and vigorous crop establishment is a key factor in achieving maximum wheat yield under the RW cropping system. Previous results indicated that the seeding rate could be efficiently increased to enhance seed germination and seedling establishment, and thereby improve fertile tiller production (Shah et al., 2020; Hussain et al., 2017; Song et al., 2016).

The nitrogen uptake of winter wheat in North China Plain was higher than that in Jianghan Plain compared with other wheat cropping areas in China (Che et al., 2016). We also observed a lower N uptake of RW compared with DW (Fig. 3). The possible reasons for the differences in N uptake between the two cropping systems may be related to the distribution of the root system in soil. Compacted soil layers highly resistant to penetration, which is one of the most common problems affecting root growth. The surface root system of RW and wheat is relatively large (Fig. 4), which increases the risk of low availability of water and nutrients to the plant. However, pre-cropping with soybean, lucerne (*Medicago sativa* L.), and chicory (*Cichorium intybus* L.) increases the density of large-size vertical biopores in the subsoil making the subsoil layers more accessible for root growth of successive crops (Köpke et al., 2015; Seidel et al., 2019). The proportion of root in 40–60 cm soil layer of DW was higher than that of RW. The deeper root growth had better access to the deep soil nitrogen source, resulting in a higher N uptake.

The soil nitrate nitrogen content at different root layers may directly reflect the nitrogen supply capacity of the upland soil to crops. We found that the nitrate nitrogen content in the shallow soil of DW was higher than that of RW (Fig. 5). Mineralization of soil nitrogen is one of the main nitrogen sources in wheat growth stage (Chen et al., 2010). The biological nitrogen fixation of soybeans increases the fertility of upland soil, resulting in a higher nitrogen accumulation in DW. The lower nitrate nitrogen content in RW may be due to: (1) the root system of RW is mainly distributed in the surface soil (0–20 cm) because of compacted soil layers, so it mainly absorbs more nitrogen at the surface; (2) in this region, soils in RW cropping system have a higher groundwater depth because of abundant seasonal rainfall during the growth period. Excessive rainfall is more likely to cause surface runoff and the impact the anaerobic environment of the rice-stubble soil. Oxygen deficiency predisposes it to denitrification and a rapid loss of nitrate (Aulakh & Bijay, 1996; Cheng et al., 2014); Additional studies should be conducted to determine effective strategies to improve nitrogen use efficiency of RW cropping system (e.g., develop high nitrogen use efficiency wheat cultivar or land drainage systems).

### Effects of nitrogen management on yield and nitrogen uptake

We found that increasing the top-dressing times significantly impacted the yield of wheat under two cropping systems. Our results showed that the difference in the spike numbers or 1,000- kernel weight was not significant between M1 and M2, and the yield advantage under M2 was mainly attributed to an increase in kernels per spike (Table 3). This suggests kernels production efficiency per unit is another key factor in achieving maximum wheat yield under the RW cropping system. Additional studies with more wheat cultivars with high kernels per spike under the RW cropping system are needed to support this claim.

The increased rainfall observed recently in the Jianghan Plain poses an additional risk for nitrate to leach from the cropland. Our results showed that increasing nitrogen supply times significantly increased nitrate nitrogen accumulation of the soil (0–200 cm) in DW, but there was no significant difference in RW (Fig. 5). This may be due to a higher soil water capacity (Fig. 2) in the paddy field, and less nitrate nitrogen leached into the deep soil with rainfall. Some N is absorbed and utilized by the crops in the season after nitrogen fertilizer is applied to the soil, some remains in the soil, and some is lost through runoff and leaching (Powlson, 1993). High soil nitrogen loss in RW may due to the following conditions: (1) the alternations of floods and droughts in the paddy field make the microbial species rich and complex, resulting in a greater consumption of soil nitrogen (Breidenbach et al., 2016); (2) the denitrification process may cause higher gaseous losses from moist soil and higher temperature conditions (Tan, Shao & Gu, 2018).

## Conclusion

Our study showed that increasing top-dressing times can narrow the grain yield gap between RW and DW, but does not improve the nitrogen use efficiency of RW. RW had lower nitrate nitrogen content in the soil surface layer (0–20 cm) and lower root length density in the deep layer (40–60 cm), which may accout for the low N uptake in RW. Our results are of interest to breeders and agronomists for improved management practices (e.g., cultivar selection and N management) and RW cropping system productivity.

##  Supplemental Information

10.7717/peerj.11189/supp-1 Supplemental Information 1 Raw data

## Additional Information and Declarations

### Data Availability

The following information was supplied regarding data availability: Raw measurements are available in the Supplementary Files.
